# Effects of ATRA on diabetic rats with renal ischemia-reperfusion injury^1^

**DOI:** 10.1590/s0102-865020200010000006

**Published:** 2020-03-27

**Authors:** Zeng Cheng, Sun Qian, Meng Qingtao, Xia Zhongyuan, Xiao Yeda

**Affiliations:** I MM, Department of Anesthesiology, People’s Hospital of Wuhan University, Wuhan, Hubei, China. Conception and design of the study, analysis and interpretation of data, manuscript preparation.; II PhD, Department of Anesthesiology, People’s Hospital of Wuhan University, Wuhan, Hubei, China. Acquisition and analysis of data.; III PhD, Department of Anesthesiology, People’s Hospital of Wuhan University, Wuhan, Hubei, China. Conception and design of the study, final approval.

**Keywords:** ATRA, Diabetes Mellitus, Ischemia, Reperfusion Injury, Rats

## Abstract

**Purpose:**

To explore the role of all-trans retinoic acid (ATRA) in renal ischemia/reperfusion injury of diabetic rats.

**Methods:**

Sixty adult male rats were randomly divided into 6 groups, including sham group (S group), ischemia-reperfusion group (I/R group), ischemia-reperfusion+ATRA group (A group), diabetic group (D group), diabetic ischemia-reperfusion group (DI/R group), diabetic ischemia-reperfusion +ATRA group (DA group). The levels of creatinine (Cr), cystatin C (Cys-C) and β2-microglobulin (β2-MG) were measured. Morphology of renal tissue was observed under light microscope.

**Results:**

DJ-1, Nrf2, HO-1 and caspase-3 were detected by western blot. DJ-1, Nrf2, HO-1 and caspase-3 in I/R group, D group and DI/R group was higher than that in S group. Compared with I/R group, Nrf2 and HO-1 in A group was decreased, but caspase-3 was increased. However, Nrf2 in DA group was higher than that in DI/R group, HO-1 and caspase-3 in DA group were lower than that in DI/R group. Compared with group S, Cr, Cys-C and β2-MG in I/R group, A group, D group, and DI/R group were higher. Whereas the levels of Cr, Cys-C, β2-MG and renal injury score in DA group were lower than those in DI/R group.

**Conclusion:**

ATRA has a protective effect on renal ischemia-reperfusion injury in diabetic rats, maybe relating to DJ/Nrf2 pathway.

## Introduction

Diabetes mellitus, as a chronic metabolic disorder, is characterized by an abnormal insulin secretion and action resulting from interaction of hereditary and environmental factors. As one of the common complications of diabetes, diabetic nephropathy has an increasing gradually incidence. The pathogenesis of diabetic nephropathy is associated with the interaction of hyperglycemia, advanced glycation end-product, increased po-lyol pathway and oxidative stress^1^. During perioperative period, renal ischemia-reperfusion injury easily occurs in diabetic patients, which aggravates diabetic nephropathy. Due to surgery, especially the complex process of kidney surgery, stress, shock and prolonged stimulation resulted in renal insufficiency^2^.

All-trans retinoic acid (ATRA) is a potent derivative of vitamin A. Studies have shown that ATRA can effectively prevent the progression of diabetes in rats. Pancreatic β-cells, acinar and ductal cells gradually restore their normal appearance under ATRA treatment. What’s more, insulin messenger RNA and serum indices almost normalize, which improves the histological changes of the pancreas and the serum indices in diabetic rats^3,4^. In addition, ATRA can affect the progression of diabetic nephropathy without causing any significant side effects and has a therapeutic effect on diabetic nephropathy^5^.

Sun *et al*.^6^have demonstrated that DJ-1/Nrf2 pathway was involved in the pathogenesis of diabetic nephropathy in rats, which played a protective role on diabetic nephropathy. Nrf2/HO-1 pathway is one of the most important endogenous antioxidant pathway^7,8^. Upregulation of Nrf2 protein levels can protect the kidneys from I/R-associated oxidative damage^9^. Meanwhile, ATRA is an inhibitor of Nrf2^10^. This study aimed to investigate whether ATRA had a protective effect on renal ischemia- reperfusion in diabetic rats and whether it was related to DJ-1/Nrf2 pathway.

## Materials

Sixty adult SD male rats, weighing 200-220g, were purchased from the Animal Experimental Center of Wuhan University. Sixty SD Rats were fed 5 days after adaptation and were randomly assigned to six groups: sham group (S group), ischemia-reperfusion group (I/R group), ischemia-reperfusion +ATRA group (A group), diabetic group (D group), diabetic ischemia reperfusion group (DI/R group), diabetic ischemia reperfusion +ATRA group (DA group), every group n =10. ATRA was purchased from Sigma (R2625-100MG), and the rats were intraperitoneally injected with 1mg/mL of ATRA at a dose of 1mL per day.

### Diabetes model

The rats were fasted overnight 12 hours before STZ injection. Then the rats received a single intraperitoneal injection of STZ (purchased from Sigma Chemical) at a dose of 60 mg/kg freshly dissolved in 0.1 M citrate buffer (ph =4.5). After 3 days of diabetes induction, tail vein blood glucose samples were collected and measured with OneTouch glucometer (Johnson & Johnson, NJ). Rats with a FBG level above16.7 mmol/L were considered diabetic and were selected^11^. Normal rats received an equivalent volume of citrate buffer only. The fasting blood glucose (FBG) level was monitored every week.

### Renal ischemia-reperfusion model12

The rats were anesthetized with pentobarbital sodium (50 mg/kg) by intraperitoneal injection. The abdomen was opened, and the bilateral renal pedicles were occluded lasting for 30 min, followed by 24 h of reperfusion.

### Measurement of serum Cr, Cys-C and β2-MG

Blood samples were collected by cardiac puncture, centrifuged at 3,000 rpm, for 10 min at 4°C. Serum was separated and stored at −20 °C. Levels of Cr, Cys-C and β2-MG were measured using ELISA assay kits (cat nos.C011-1, E-EL-R0304c and E-EL-R1085c; Elabscience Biotechnology Co., Ltd., Wuhan, China) according to the manufacturer’s instructions.

### Histological examination

The left kidney was cut into sections and fixed in 4% formaldehyde, embedded in paraffin and sectioned into 4 μm slices for light microscopy (original magnification, ×200; Olympus BX50; Olympus Corporation), followed by staining with hematoxylin and eosin (HE). Histological assessment of tubular necrosis was semi-quantitatively determined using a method modified from McWhinnie *et al*.^13^using the following scoring system: 0), normal histology; 1), tubular cell swelling, brush border loss and nuclear condensation, with up to one-third of the tubular profile exhibiting nuclear loss; 2), same as for score 1, but more than one-third and less than two-thirds of the tubular profile displaying nuclear loss; and 3), same as for score 1, but more than two-thirds of the tubular profile exhibiting nuclear loss.

### Western blot analysis

Western blot analysis was performed as previously described^14,15^. Briefly, equal amounts of protein (50 µg) were separated by 12% SDS-PAGE at 100 V for 3 h. After electrophoresis, proteins were transferred onto polyvinylidenedifluoride membranes at 200 mA for 70 min. The membranes were incubated with the primary antibodies (against active DJ-1, Nrf2, HO-1, caspase-3). After washing three times in TBS-T, membranes were incubated with anti-rabbit immunoglobulin G conjugated to horseradish peroxidase at a dilution of 1: 2,000 in TBS-T containing 5 % skimmed milk for 2 h at room temperature. The immunoreactive bands were visualized by enhanced chemiluminescence (PerkinElmer, Inc., Waltham, MA, USA) and captured on X-ray film. Blots were stained with an anti-β-actin antibody, and the protein levels were normalized with respect to β-actin band density.

### Statistical analysis

All data are expressed as the mean ±standard deviation (SD). Statistical analysis was performed using GraphPad Prism software version 7.0 (GraphPad Software, Inc., La Jolla, CA, USA). Analysis of variance (ANOVA) test and Student’s t test were used to evaluate statistical significance. The level of significance was set at P<0.05 for all statistical tests.

## Results

### Renal histopathology

**Figure 1:**
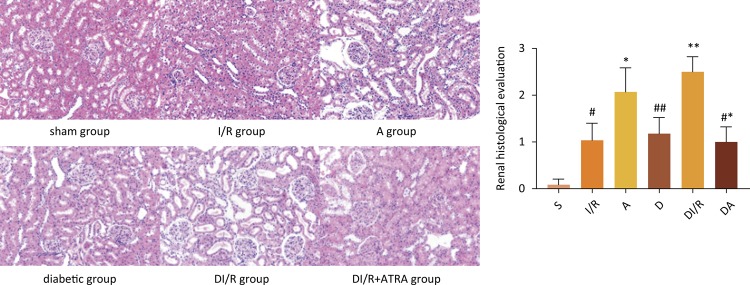
- Renal histological evaluation of kidney injury and quantitative injury scores (hematoxylin and eosin staining; magnification, ×200). Data are expressed as the mean ± standard deviation (n=10). #*P*<0.05 *vs*. sham group, *P<0.05 *vs*. I/R group, ##*P*<0.05 *vs*. sham group, **P<0.05 *vs*. D group, #*P<0.05 *vs*. DI/R group.

Edema, tubular cell necrosis and cytoplasmic vacuoles were observed in histological specimens in I/R group, A group, D group and DI/R group, but were absent in S group. Compared with the renal histological evaluation score of kidneys obtained from S group, I/R group and D group exhibited a clearly increase in renal histologic evaluation score (*P*<0.05). This increase was significantly enhanced in I/R group and DI/R group compared to I/R group and D group individually respectively (*P*<0.05). Histological alterations were obviously improved in specimens from DA group compared to DI/R group (Fig. 1, *P*<0.05).

### Serum level of Cr, Cys-C and β2-MG

**Figure 2:**
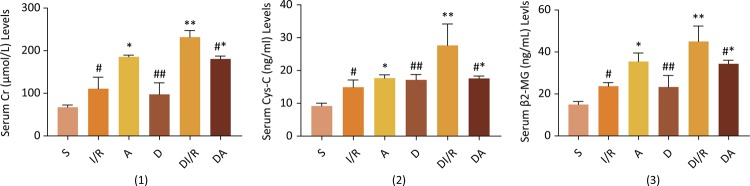
- Serum level of Cr, Cys-C and β2-MG. (1) Cr:S group 67.27±5.46 μmol, I/R group: 111.00±26.97 μmol, A group: 185.5±4.20 μmol, D group: 97.56±27.07 μmol, DI/R group: 231.84±15.57 μmol, DA group: 180.50±6.76 μmol; (2) Cys-C: S group: 9.18±0.88 ng, I/R group: 14.92±2.19 ng, A group: 17.67±1.02 ng, D group: 17.14±1.64 ng, DI/R group: 27.66±6.53 ng, DA group: 17.57±0.74 ng; (3) β2-MG: S group: 14.99±1.41 ng, I/R group: 23.73±1.68 ng, A group: 35.46±4.08 ng, D group: 23.32±5.4 8 ng, DI/R group: 45.01±7.33 ng, DA group: 34.36±1.72 ng. Data are expressed as the mean ± standard deviation (n=10). #*P*<0.05 *vs*. sham group, *P<0.05 *vs*. I/R group, ##*P*<0.05 *vs*. sham group, **P<0.05 *vs*. D group, #*P<0.05 *v*s. DI/R group.

The serum levels of Cr, Cys-C and β2-MG in I/R group and D group were significantly higher than in S group (*P*<0.05). And those blood indices were increased furtherly in A group and DI/R group compared to I/R group and D group individually (*P*<0.05). Administration of ATRA visibly reduced the Cr, Cys-C and β2-MG levels compared with DI/R group rats (Fig. 2, *P*<0.05).

### The expression of DJ-1, Nrf2, HO-1 and caspase-3 in renal tissues

**Figure 3:**
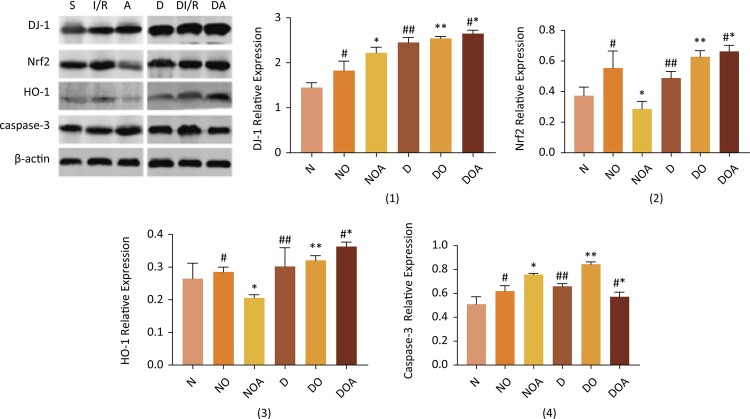
The protein expression of DJ-1, Nrf2, HO-1 and caspase-3. As examined by western blotting, and proteins level DJ-1, Nrf2, HO-1 and caspase-3 in the renal tissue. Data are expressed as the mean ± standard deviation (n=10). #*P*<0.05 *vs*. sham group, *P<0.05 *vs*. I/R group, ##*P*<0.05 *vs*. sham group, **P<0.05 *vs*. D group, #*P<0.05 *vs*. DI/R group.

Compared with sham group, the expression of DJ-1, Nrf2, HO-1 and caspase-3 in I/R group and D group were increased remarkably. And administration of ATRA significantly decreased the expression of Nrf2 and HO-1 in A group contrasted with I/R group. Moreover, the expression of DJ-1 and caspase-3 in A group strengthened much more than in I/R group. In DI/R group, the expression of DJ-1, Nrf2, HO-1 and caspase-3 also were enhanced in contrast with D group. However, the expression of Nrf2 and HO-1 in DA group was heightened compared with DI/R group, and the caspase-3 expression in DA group reduced perceptibly (Fig. 3, *P*<0.05).

## Discussion

The present study found that ATRA inhibited the expression of Nrf2 leading to a decreasing of HO-1 in A group, but the expression of caspase-3, the serum levels of Cr, Cys-C and β2-MG and the renal histological evaluation score of kidneys were increased in A group more than those in I/R group. It demonstrated that ATRA aggravated kidney damage caused by renal ischemia-reperfusion. However, when ATRA was administrated in diabetic rats with ischemia-reperfusion, the expression of Nrf2 strengthened while the expression of caspase-3, the serum levels of Cr, Cys-C and β2-MG and the renal histological evaluation score of kidneys was decreased in comparison with DI/R group. Thus, ATRA not only improved the histological alterations and the blood indices, but also ameliorated kidney function and mitigated kidney damage.

ATRA exerts beneficial effects on nephropathy^16-18^. ATRA improves the histological changes of the pancreas of diabetic rats resulting in a significant decrease in blood glucose and total peroxides, elevated pancreatic insulin mRNA, and serum insulin^4^. ATRA shows to limit glomerular cell proliferation and kidney damage by reducing renal TGF-β1 and TGF receptor II expression in nephropathy rat models^16^. ATRA reduces the incidence of T1DM by protecting islet β-cells, upregulating the levels of the pancreatic β-cell factor IL-4, reducing the cell toxicity-associated levels of IFN-γ in β-cells, and maintaining the Th1/Th2 balance^3^. What’s more, ATRA exhibits an anti-oxidant property by blocking lipid peroxidation in streptozotocin-induced diabetic rats^19^. It has been suggested that oxidative stress, as a key factor, is involved in the pathogenesis and progression of diabetic nephropathy (DN)^20-22^. DJ-1/Nrf2 pathway has been found to perform a protective effect on diabetic nephropathy^6,23^, which is one of the most important endogenous antioxidant pathway^7,8^. In this study, the expression of DJ-1/Nrf2/HO-1 in DA group was upregulated, and histological alterations clearly improved edema, as tubular cell necrosis and cytoplasmic vacuoles almost disappeared. These data suggested that ATRA exerted protective effects in STZ-induced diabetic rats with renal ischemia-reperfusion injury.

In the renal ischemia-reperfusion rats model, increased oxidative stress also participates in the pathogenesis of renal I/R and deteriorates kidney injury^24,25^. Nrf2 is known as the “master regulator” of antioxidant/anti-inflammatory/cytoprotective responses^26-28^. Under normal conditions, Nrf2 is retained in the cytoplasm, being bound to KEAP1, which targets it for ubiquination and proteosomal destruction^27-29^. In response to oxidative stress, critical KEAP1 cysteine residues are disrupted, Nrf2 is thus freed from ubiquination, and enhanced nuclear translocation results. In concert with a small Maf protein, and with export of the nuclear repressor Bach1, Nrf2 binds to antioxidant response elements (AREs) in the promoter regions of diverse cytoprotective genes. However, ATRA is an inhibitor of transcription factor Nrf2 by interfering with recruitment of Nrf2 to the ARE, whereas nuclear levels of Nrf2 are not affected by ATRA^10^. In this study, the expression of Nrf2/HO-1 in I/R group was decreased, but the expression of caspase-3 was increased, which is related to apoptosis^30^. Otherwise, the kidney injury scores and the blood indices in I/R group were worsen. These showed that ATRA indeed limited the expression of Nrf2, leading to increased apoptosis and aggravated kidney injury.

Taken together, ATRA impacts the kidney histopathology and function in renal ischemia-reperfusion rat model by downregulating Nrf2 protein expression. However, in STZ-induced diabetic rats with renal ischemia-reperfusion injury, due to many renal protective factors of ATRA as above stated, the effect of inhibiting Nrf2 does not perform a central role. Conversely, accompanying with the expression of DJ-1 upregulated, Nrf2 protein expression also increased so that more Nrf2 exerted an anti-oxidant effect. The deficiency of this study is that it does not explore the relationship between ATRA and the DJ-1/Nrf2 pathway, and that the mechanism of ATRA renal protective in STZ-induced diabetic rats with renal ischemia-reperfusion injury is still unclear.

## Conclusion

This study suggested that ATRA played a protective effect on renal ischemia-reperfusion injury in diabetic rats, which might relate to the DJ/Nrf2 pathway. Our results might provide a potential therapeutic strategy against diabetic renal ischemia-reperfusion injury.

## Footnotes

1 Research performed at Department of Anesthesiology, People’s Hospital of Wuhan University, Wuhan, Hubei, China.
